# The associations between weight-related anthropometrics during childhood and lung function in late childhood: a retrospective cohort study

**DOI:** 10.1186/s12890-017-0567-3

**Published:** 2018-01-19

**Authors:** Kristine Kjer Byberg, Ingvild Bruun Mikalsen, Geir Egil Eide, Michele R. Forman, Pétur Benedikt Júlíusson, Knut Øymar

**Affiliations:** 1 Department of Paediatrics, Stavanger University Hospital, POB 8100, N-4068 Stavanger, Norway; 2 Department of Clinical Science, University of Bergen, Bergen, Norway; 3 Centre for Clinical Research, Haukeland University Hospital, Bergen, Norway; 4 Department of Global Public Health and Primary Care, University of Bergen, Bergen, Norway; 5 Department of Nutrition Science, Purdue University, West Lafayette, IN USA; 6 Department of Paediatrics, Haukeland University Hospital, Bergen, Norway

**Keywords:** Birth weight, Body mass index, Skin fold thickness, Lung function tests, Child

## Abstract

**Background:**

An association between body weight in childhood and subsequent lung function and asthma has been suggested, but few longitudinal studies exist. Our aim was to explore whether weight-related anthropometric measurements through childhood were associated with lung function in late childhood.

**Methods:**

From an original nested case-control study, a cohort study was conducted, where lung function was measured in 463 children aged 12.8 years, and anthropometry was measured at several ages from birth through 12.8 years of age. Associations between anthropometrics and lung function were analysed using multiple linear and fractional polynomial regression analysis.

**Results:**

Birthweight and body mass index (BMI; kg/m^2^) at different ages through childhood were positively associated with forced vital capacity in percent of predicted (FVC %) and forced expiratory volume in the first second in percent of predicted (FEV_1_%) at 12.8 years of age. BMI, waist circumference, waist-to-height ratio and skinfolds at 12.8 years of age and the change in BMI from early to late childhood were positively associated with FVC % and FEV_1_% and negatively associated with FEV_1_/FVC and forced expiratory flow at 25–75% of FVC/FVC. Interaction analyses showed that positive associations between anthropometrics other than BMI and lung function were mainly found in girls. Inverse U-shaped associations were found between BMI at the ages of 10.8/11.8 (girls/boys) and 12.8 years (both genders) and FVC % and FEV_1_% at 12.8 years of age.

**Conclusions:**

Weight-related anthropometrics through childhood may influence lung function in late childhood. These findings may be physiological or associated with air flow limitation. Inverse U-shaped associations suggest a differential impact on lung function in normal-weight and overweight children.

**Trial registration:**

This study was observational without any health care intervention for the participants. Therefore, no trial registration number is available.

**Electronic supplementary material:**

The online version of this article (doi: 10.1186/s12890-017-0567-3) contains supplementary material, which is available to authorized users.

## Background

Birth weight is positively associated with subsequent lung function in both childhood and adulthood [1–3]. This agrees with Barker’s results, suggesting that an adverse environment and poor growth in utero may lead to impaired growth of airways and subsequent reduced airway calibre [4].

In adulthood, body mass index (BMI) and different measures of body composition are inversely associated with lung function [5, 6]; however, in childhood, these findings are less consistent. Cross-sectional studies of children and adolescents report positive associations between BMI or other anthropometric measures and forced vital capacity (FVC) and forced expiratory volume in the first second (FEV_1_), but negative associations with FEV_1_/FVC [7–10]. The impact of catch-up growth and change in weight over time on lung function in later childhood has been studied in longitudinal studies, with inconsistent results reported at different ages [11–13]. Moreover, the age at which a possible transition occurs from the initial positive to the negative associations between anthropometrics and lung function is not known.

Studies including obese children have shown a reduction in static lung volume related to the degree of obesity [14] and inverse associations between waist circumference, skinfolds and lung function [8], but study results were inconsistent [15]. Overweight/obesity has been associated with childhood asthma; however, the causal pathway for this relationship remains unknown [16].

BMI is a surrogate measure of body fat and reflects total body mass; however, it does not provide information about fat distribution, and it may misclassify persons with well-developed musculature and children with low to normal BMI [17]. Body weight and BMI do not distinguish between fat mass and lean (muscle) mass, which may have opposite effects on lung function [18]. Therefore, other weight-related anthropometrics like waist circumference and skinfolds have been suggested as markers of body fat distribution [19–21]. The impact of waist circumference and skinfolds on lung function during childhood has not been studied in detail.

The present cohort study was derived from a case-control study nested within a birth cohort. The aim was to study the associations between birthweight, BMI, changes in BMI, waist circumference, triceps- and subscapular skinfolds during childhood on lung function at 12.8 years of age. We hypothesized that childhood overweight or accelerated weight gain is associated with an obstructive lung function pattern in late childhood.

## Methods

### Study population and design (Fig. 1)

**Fig. 1:**
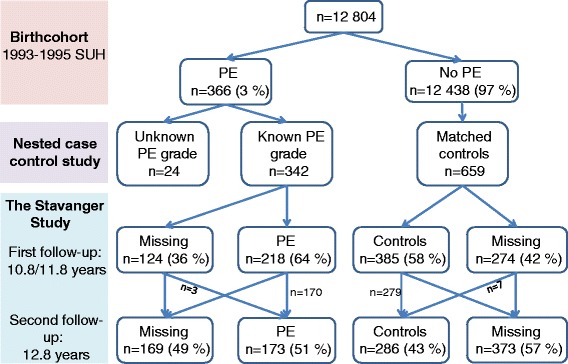
The origin and design of the Stavanger study. The percentages in the second line are relative to the total birth cohort. The percentages shown for the Stavanger Study are relative to the numbers with known grade of PE and matched controls in the nested case-control study. Abbreviations: SUH = Stavanger University Hospital; PE = preeclampsia; n = number of participants

The study was a part of the ‘Stavanger study’ which is described in detail previously [22]. A flow-chart presenting number of participants and loss to follow-up is shown in Fig. 1. All children born at Stavanger University Hospital in 1993–1995 (*n* = 12,804) comprised a population-based birth cohort. From this cohort, a nested case-control study of preeclampsia exposure for perinatal risks was conducted, where offspring exposed to maternal preeclampsia and unexposed offspring were identified as follows: For each exposed offspring, two matched unexposed offspring were selected: one was defined as the next born in the hospital (i.e. a birth date and gender match) and one as the next born matched on maternal age (i.e. a risk factor for preeclampsia). Children without adequate information about preeclampsia severity were excluded from analyses [23]. The ‘Stavanger study’ was conducted as a follow-up of the nested case-control study, where the children were invited to participate in two follow-ups in late childhood: a first follow-up at the target ages of 10.8 years (girls) and 11.8 years (boys), and a second follow-up at the target age of 12.8 years (both genders) [24]. The ages for the first and second follow-ups were selected to coincide with the age of pubertal onset of Norwegian children and the age of menarche of the girls respectively. In the current study, data from the follow-up of the nested case-control study were treated as those of a retrospective cohort study, and to avoid bias, all analyses were adjusted for preeclampsia. Owing to missing participants, the original matching of maternal age and birth date was ignored in the analyses; therefore, all analyses were adjusted for maternal age. All children with information about preeclampsia severity who participated in lung function testing at the second follow-up formed the analytic sample.

The study was approved by the Norwegian Data Inspectorate, the Regional Committee for Ethics in Medical Research for western Norway, and the Institutional Review Boards of the National Cancer Institute and University of Texas at Austin, United States. At follow-up, mothers and children signed an informed consent/assent form.

### Outcomes recorded at 12.8 years of age

Lung function was measured by spirometry according to established guidelines [25] by using a Vmax Encore spirometer (Sensor Medics Inc., Anaheim, USA), and FVC, FEV_1_ and forced expiratory flow at 25–75% of FVC (FEF_25–75%_) were recorded. Measurements were compared to values predicted by standard reference equations [26] and reported as percentages of predicted (FVC %, FEV_1_%, and FEV_1_/FVC %). The FEF_25–75%_/FVC ratio was given as a percentage as no predicted values were available.

### Predictors

Birthweight was abstracted from hospital records. Recordings of length/height and weight measurements were collected from well-child clinics at the target ages of 3, 6 and 12 months and 4 years. Trained nurse researchers measured the height, weight, triceps skinfold and waist circumference twice in offspring at both follow-ups at the ages of 10.8/11.8 (girls/boys) and 12.8 years (both genders). The subscapular skinfold was measured twice at the second follow-up [22, 27]. At the follow-ups, but not at birth or at the well-child clinics, the average of two measurements was used in the analysis. The waist-to-height ratio was calculated as the waist circumference divided by height.

Change in weight and BMI standard deviation scores (SDS) was calculated as the difference in weight and BMI SDS from each target age to the next. Weight status was classified as an ordinal variable with 6 levels: thinness grades 1, 2 and 3, normal-weight, overweight and obese, by comparing BMI with age- and sex-specific cut-off values of the International Obesity Task Force (IOTF) [28]. Accelerated weight gain was defined as an increase in BMI SDS between two target ages.

At 12.8 years of age, the children answered a validated questionnaire from the International Study of Asthma and Allergy in Childhood (ISAAC) recording symptoms of asthma [29]. Current asthma was defined as a positive answer to ‘asthma ever’ and a positive answer to at least one of the two questions on wheezing/whistling/chest tightness or the use of asthma medication during the last 12 months. Missing answers were interpreted as negative.

### Potential confounders

Mothers completed a questionnaire at the first follow-up with information about birth order, breastfeeding, mother’s doctor’s diagnosis of asthma and mother’s education. Information about gestational age was abstracted from hospital records. Potential confounders included the following categorical and continuous variables: gender, birth order (firstborn or later), gestational age (weeks), duration of breastfeeding (categories: none, < 3 months, > 3 months), mother’s preeclampsia (none, mild/moderate, severe) [30], mother’s BMI (weight at first antenatal visit and height at the first follow-up), mother’s smoking (at first antenatal visit), mother’s doctor-diagnosed asthma, mother’s education (< 9 years, 9–12 years, > 12 years) and mother’s age [24]. Lung function measurements were reported as percentages of predicted values standardized according to age, height, gender and ethnicity. Therefore, age and height were not included as potential confounders.

At the first follow-up at age 10.8/11.8 years (girls/boys), the ‘Stanford Brief Activity Survey’, a questionnaire validated for adults, was administered to the mothers for responses about the child’s physical activity [31]. Specifically, the answers to the following questions were obtained: ‘How active was your child at 3–6 years?’ and ‘How active was your child at 6–10 years?’ The response categories were categorized as passive and/or not so active = low activity, active = normal, very active = high activity.

For the analyses of the association between changes in weight/BMI SDS and lung function, the weight/BMI SDS before each change was included as a possible confounder.

If child’s asthma is causally related to anthropometrics and/or lung function, the direction of causality may go either way. Therefore, it was not included as a confounder. The confounders are illustrated in a Directed Acyclic Graph (Additional file 1: Figure S1) [32].

### Statistics

As the lung function measurements were added as outcomes in an established follow-up study, power calculations were not performed prior to study start.

For normally distributed variables, means with standard deviations for descriptive statistics and Gosset’s unpaired t-test for group comparisons were calculated.

There was a wide range of gestational ages at birth owing to the inclusion of offspring of preeclampsia and normotensives and a wide age range at later well-child visits, and analysing actual values for anthropometrics as predictors would not be appropriate. Therefore, standard deviation scores (SDS) based on anthropometric values and actual ages were computed from relevant references [20, 21, 33, 34]. Conversions into SDS were performed using R version 2.6.2 (R Development Core Team, Vienna, Austria).

The associations between BMI SDS and outcomes of lung function at 12.8 years were analysed by multiple linear regression analyses. Separate analyses of the predictors were performed for each target age, for each change in weight/BMI SDS in early childhood and both follow-ups. Each variable was entered separately into simple regression models. Next, all potential confounders were included in fully adjusted models. Thereafter, we tested for interactions between predictors and gender. Owing to missing values, the number of participants varied between the different analyses.

For each predictor, estimated coefficients (b) with F-test *P*-values and 95% confidence interval (CI) are reported. The significance level was chosen as 0.05 for all tests.

To study possible non-straight-line associations between BMI SDS and the outcomes, multiple fractional polynomial regression analysis (MFPR) was used with the requirement of *p* ≤ 0.05 for non-straight-line terms. Finally, separate analyses were performed for each gender.

IBM SPSS for Windows (Version 22.0.0, Chicago, Ill., USA) was used for descriptive statistics and linear regression, and Stata SE 14 was used for MFPR.

## Results

### Characteristics of the participants (Tables 1, 2 and 3)

In total, 468 children consented to participate at the second follow-up at 12.8 years of age, but information about preeclampsia severity was available for only 459 children (173 exposed to preeclampsia and 286 unexposed). Successful lung function measurement was performed in 453 of these children, and they constituted the analytic sample. Of these, 446 children also completed the ISAAC questionnaire. The number of anthropometric measures from each target age differs owing to missing data from the well-child visits. The response rate for each potential confounder varies owing to missing data from the questionnaires and antenatal visits.

**Table 1:** Clinical characteristics of 463 Norwegian children delivered at Stavanger University Hospital in Norway 1993–1995 according to anthropometrics predictors

Predictor	n	Mean	SD	SDS mean	SD of SDS
Anthropometrics					
Birthweight (kg)	452	3.36	0.73	−0.20	1.22
BMI 3 months	419	16.4	1.52	0.04	0.99
BMI 6 months	434	17.2	1.56	0.12	1.06
BMI 1 year	433	17.2	1.51	0.21	1.10
BMI 4 years	386	15.9	1.38	−0.08	1.06
BMI 10.8/11.8 years^a^	442	17.8	2.81	−0.21	1.16
BMI 12.8 years	453	18.8	2.96	−0.10	1.17
WC					
10.8/11.8 years^a^	442	63.0	7.49	0.07	1.08
12.8 years	453	68.2	7.75	0.45	1.01
WHtR					
10.8/11.8 years^a^	442	0.42	0.04	0.05	1.11
12.8 years	453	0.43	0.05	0.45	0.99
TSF					
10.8/11.8 years^a^	439	11.5	4.50	−0.27	1.11
12.8 years	452	12.2	4.86	−0.14	1.06
SSF					
12.8 years	437	8.34	3.93	−0.35	1.01
Change in weight/BMI		Change in weight (kg) or BMI	SD	Change in SDS	SD of SDS
Birthweight–3 months (kg)	430	2.71	0.71	0.11	1.26
BMI 3–6 months	418	0.82	1.08	0.09	0.74
BMI 6–12 months	431	−0.02	1.09	0.09	0.77
BMI 1–4 years	383	−1.38	1.39	−0.31	1.01
BMI 4–10.8/11.8 years^a^	381	1.90	2.48	−0.14	1.10
BMI 10.8/11.8^a^–12.8 years	442	1.07	1.52	0.11	0.67

*Abbreviations: SD* standard deviation, *SDS* SD score, *BMI*, body mass index (kg/m^2^), *WC* waist circumference (cm), *WHtR* waist-to-height ratio, *TSF* triceps skinfold (mm), *SSF* subscapular skinfold (mm)

^a^10.8 years for girls, 11.8 years for boys

**Table 2:** The distribution of extended IOTF BMI classes for the study cohort of children delivered at Stavanger University Hospital in Norway 1993–95 at 4, 10.8/11.8^a^ and 12.8 years of age

Extended IOTF BMI class	4 years of age *n* = 375	10.8/11.8 years of age^a^ *n* = 442	12.8 years of age *n* = 453
	n (%)	n (%)	n (%)
Thinness grade 3	1 (0.3)	3 (0.7)	6 (1.3)
Thinness grade 2	6 (1.6)	11 (2.5)	9 (2.0)
Thinness grade 1	31 (8.3)	52 (11.8)	47 (10.4)
Normal weight	297 (79.2)	315 (71.3)	330 (72.8)
Overweight	35 (9.3)	51 (11.5)	50 (11.0)
Obese	5 (1.3)	10 (2.3)	11 (2.4)

*Abbreviations: IOTF* International Obesity Task Force, *BMI* body mass index

^a^10.8 years for girls, 11.8 years for boys

**Table 3:** Comparisons of different lung function variables at 12.8 years for the study cohort of children without and with asthma who were delivered at Stavanger University Hospital in Norway in 1993–1995

Lung function measure	All *n* = 453^a^	No asthma *n* = 409	Current asthma *n* = 37	T-test *p*-value
	Mean (SD)	Mean (SD)	Mean (SD)	
FVC %	100.1 (11.6)	100.2 (11.8)	101.1 (9.5)	0.658
FEV1%	97.5 (10.7)	97.6 (10.8)	96.2 (10.0)	0.456
FEV1/FVC %	97.1 (7.5)	97.2 (7.5)	94.9 (7.6)	0.069
FEF_25–75%_ /FVC	93.0 (23.5)	93.7 (23.7)	83.5 (20.8)	0.012

*Abbreviations: SD* standard deviation, *FVC %* forced vital capacity in percent of predicted, *FEV1%* forced expiratory volume in first second in percent of predicted, *FEV1/FVC %* ratio of FEV1 over FVC in percent of predicted, *FEF*_*25–75%*_*/FVC* ratio of forced expiratory flow between 25% and 75% of the forced vital capacity over FVC given as a percentage value

^a^Information on asthma diagnosis was missing for 7 children

Baseline characteristics were similar between those who assented to the first follow-up only and those who assented to both follow-ups [24]. The mothers of the children who assented to the second follow-up were older than the mothers of the children who did not assent. Otherwise, perinatal characteristics were similar between those who assented or did not assent to both follow-ups [35]. The available clinical characteristics of the 463 children who performed lung function tests are shown in Table 1. The number of children in the different IOTF weight classes at 4 years of age and both follow-ups is shown in Table 2. Compared to children without asthma, children with current asthma had similar FVC % and FEV_1_%, lower FEF_25–75%_/FVC and a tendency for lower FEV_1_/FVC % (Table 3).

### Anthropometrics and lung function (Tables 4 and 5 and Fig. 2)

Birthweight and BMI SDS at all ages were positively associated with FVC % and FEV_1_% at 12.8 years of age. BMI SDS at the ages of 10.8/11.8 (girls/boys) and 12.8 years (both genders) was negatively associated with FEV_1_/FVC % and FEF_25–75%_/FVC at 12.8 years of age (Table 4).

**Table 4:** Summary of adjusted linear regression analyses of lung function at 12.8 years in 463 Norwegian children according to weight-related anthropometrics

	Outcome variable^a^												
		FVC %			FEV1%			FEV_1_/FVC %			FEF_25–75%_/FVC		
Predictor	n	b	95% CI	F-test p	b	95% CI	F-test p	b	95% CI	F-test p	b	95% CI	F-test p
Weight SDS													
Birth	370	1.54	(0.51, 2.58)	**0.004**	1.60	(0.63, 2.56)	**0.001**	0.06	(−0.62, 0.75)	0.855	−0.67	(−2.77, 1.43)	0.529
BMI SDS													
3 months	346	1.99	(0.72, 3.25)	**0.002**	1.56	(0.37, 2.75)	**0.011**	−0.50	(−1.34, 0.34)	0.242	−0.98	(−3.54, 1.57)	0.448
6 months	347	1.60	(0.42, 2.78)	**0.008**	1.43	(0.33, 2.53)	**0.011**	−0.29	(−1.08, 0.50)	0.471	−0.59	(−3.00, 1.82)	0.629
1 year	345	1.51	(0.39, 2.62)	**0.008**	1.03	(−0.02, 2.07)	0.055	−0.54	(−1.28, 0.21)	0.157	−1.06	(−3.33, 1.21)	0.358
4 years^b^	303	2.42	(1.17, 3.66)	**<0.001**	1.92	(0.75, 3.09)	**0.001**	−0.57	(−1.42, 0.29)	0.193	−1.14	(−3.71, 1.42)	0.381
10.8/11.8 years^c, d^	351	3.40	(2.36, 4.43)	**<0.001**	1.98	(0.98, 2.98)	**<0.001**	−1.47	(−2.18, −0.76)	**<0.001**	−2.87	(−5.06, −0.67)	**0.011**
12.8 years^c^	351	2.84	(1.79, 3.88)	**<0.001**	1.49	(0.49, 2.50)	**0.004**	−1.39	(−2.09, −0.69)	**<0.001**	−3.17	(−5.35, −1.00)	**0.004**
Other anthropometric SDS^c^													
WC													
10.8/11.8 years^d^	351			**0.018**			**0.010**	−1.18	(−1.95, −0.41)	**0.003**	−2.83	(−5.20, −0.46)	**0.019**
Girls	177	3.88	(2.31, 5.46)		2.83	(1.33, 4.33)		n.a.	n.a.		n.a.	n.a.	
Boys	174	1.24	(−0.33, 2.82)		0.12	(−1.38, 1.62)		n.a.	n.a.		n.a.	n.a.	
12.8 years	351			**0.009**			**0.029**	−1.23	(−2.05, −0.41)	**0.003**	−3.03	(−5.55, −0.51)	**0.019**
Girls	177	4.82	(3.24, 6.40)		3.36	(1.84, 4.88)		n.a.	n.a.		n.a.	n.a.	
Boys	174	1.75	(0.02, 3.48)		0.90	(−0.76, 2.56)		n.a.	n.a.		n.a.	n.a.	
WHtR													
10.8/11.8 years^d^	351	2.13	(0.99, 3.27)	**<0.001**	0.66	(−0.43, 1.74)	0.234	−1.52	(−2.27, −0.77)	**<0.001**	−2.58	(−4.91, −0.26)	**0.030**
12.8 years	351			**0.016**	1.45	(0.26, 2.63)	**0.017**	−1.82	(−2.64, −0.99)	**<0.001**	−3.15	(−5.72, −0.58)	**0.017**
Girls	177	4.66	(2.98, 6.34)		n.a.	n.a.		n.a.	n.a.		n.a.	n.a.	
Boys	174	1.76	(0.04, 3.48)		n.a.	n.a.		n.a.	n.a.		n.a.	n.a.	
TSF													
10.8/11.8 years^d^	349	0.31	(−0.84, 1.45)	0.597	−0.21	(−1.28, 0.86)	0.696	−0.63	(−1.38, 0.13)	0.103	−0.11	(−2.42, 2.20)	0.926
12.8 years	350			**0.005**			**0.001**	−0.59	(−1.37, 0.19)	0.136	−0.10	(−2.47, 2.28)	0.937
Girls	176	2.19	(0.49, 3.88)		1.87	(0.29, 3.44)		n.a.	n.a.		n.a.	n.a.	
Boys	174	−1.12	(−2.70, 0.45)		−1.64	(−3.10, −0.17)		n.a.	n.a.		n.a.	n.a.	
SSF													
12.8 years	340			**0.035**	1.01	(−0.23, 2.24)	0.110	−1.08	(−1.95, −0.20)	**0.016**	−1.26	(−3.95, 1.44)	0.360
Girls	175	3.27	(1.52, 5.02)		n.a.	n.a.		n.a.	n.a.		n.a.	n.a.	
Boys	165	0.65	(−1.17, 2.46)		n.a.	n.a.		n.a.	n.a.		n.a.	n.a.	

Statistically significant p-values are indicated in bold numbers. Where a significant interaction with gender was found, the *p*-value of the interaction and the coefficients and confidence intervals given each gender are presented

*Abbreviations: FVC %* forced vital capacity in percent of predicted, *FEV1%* forced expiratory volume in first second in percent of predicted, *FEV1/FVC %* ratio of FEV_1_ over FVC in percent of predicted, *FEF*_*25–75%*_*/FVC* ratio of forced expiratory flow between 25% and 75% of the forced vital capacity over FVC given as a percentage value, *n* number of participants; b: regression coefficient, *CI* confidence interval, *BMI* body mass index (kg/m^2^), *SDS* standard deviation score, *F-test p* refers to exposure only, *WC* waist circumference (cm), *WHtR* waist-to-height ratio, *TSF* triceps skinfold (mm), *SSF* subscapular skinfold (mm), *n.a.* not applicable

^a^Model adjusted for sex, birth order, gestational age, duration of breastfeeding (none, < 3 months, > 3 months) (not for weight SDS at birth, and for BMI SDS at 3 months), preeclampsia (none/mild or moderate/severe), maternal BMI and maternal smoking at first antenatal visit (yes/no), maternal age, maternal asthma and maternal education

^b^Also adjusted for physical activity at 3–6 years

^c^Also adjusted for physical activity at 6–10 years

^d^10.8/11.8 (girls/boys) years of age

**Table 5:** Summary of adjusted linear regression analyses of lung function at 12.8 years in 463 Norwegian children according to change in weight and BMI SDS

	Outcome variable^a^												
		FVC %			FEV_1_%			FEV_1_/FVC %			FEF _25–75%_/FVC		
Predictor	n	b	95% CI	F-test p	b	95% CI	F-test p	b	95% CI	F-test p	b	95% CI	F-test p
Weight SDS													
Birth–3 months	356	0.26	(−0.84, 1.36)	0.642	0.32	(−0.71, 1.35)	0.540	−0.01	(−0.74, 0.72)	0.974	−0.28	(−2.51, 1.96)	0.808
BMI SDS													
3–6 months	345	0.84	(−0.94, 2.62)	0.356	0.50	(−1.17, 2.17)	0.557	−0.47	(−1.65, 0.71)	0.434	−1.07	(−4.66, 2.52)	0.557
6–12 months	344	0.89	(−0.77, 2.55)	0.292	0.02	(−1.54, 1.58)	0.980	−0.83	(−1.94, 0.29)	0.145	−1.71	(−5.10, 1.68)	0.321
1–4 years	303	1.82	(0.27, 3.36)	**0.021**	1.82	(0.36, 3.29)	**0.015**	−0.08	(−1.13, 0.98)	0.884	0.11	(−3.06, 3.28)	0.945
4–10.8/11.8 years^b, c^	303	2.81	(1.47, 4.15)	**<0.001**	1.21	(−0.08, 2.50)	0.065	−1.64	(−2.57, −0.72)	**0.001**	−3.31	(−6.13, −0.49)	**0.021**
10.8/11.8^c^ − 12.8 years^d^	351	0.33	(−1.42, 2.07)	0.712	−0.26	(−1.95, 1.43)	0.761	−0.62	(−1.81, 0.57)	0.308	−2.52	(−6.21, 1.17)	0.180

Statistically significant *p*-values are indicated in bold numbers

*Abbreviations: FVC %* forced vital capacity in percent of predicted, *FEV1%* forced expiratory volume in first second in percent of predicted, *FEV1/FVC %* Ratio of FEV over FVC in percent of predicted, *FEF*_*25–75%*_*/FVC* forced expiratory flow between 25% and 75% of the forced vital capacity over FVC given as a percentage; n: number of participants, *b* regression coefficient, *CI* Confidence interval, *BMI* body mass index (kg/m^2^), *SDS* standard deviation score, *F-test p* refers to exposure only

^a^Model adjusted for sex, body size at start of interval, birth order, gestational age, duration of breastfeeding (none, < 3 months, > 3 months) (not for weight SDS at birth, and for BMI SDS at 3 months), preeclampsia (none/mild or moderate/severe), maternal BMI and maternal smoking at first antenatal visit (yes/no), maternal age, maternal asthma, maternal education

^b^Also adjusted for physical activity at 3–6 years

^c^10.8/11.8 (girls/boys) years of age

^d^Also adjusted for physical activity at 6–10 years of age

**Fig. 2:**
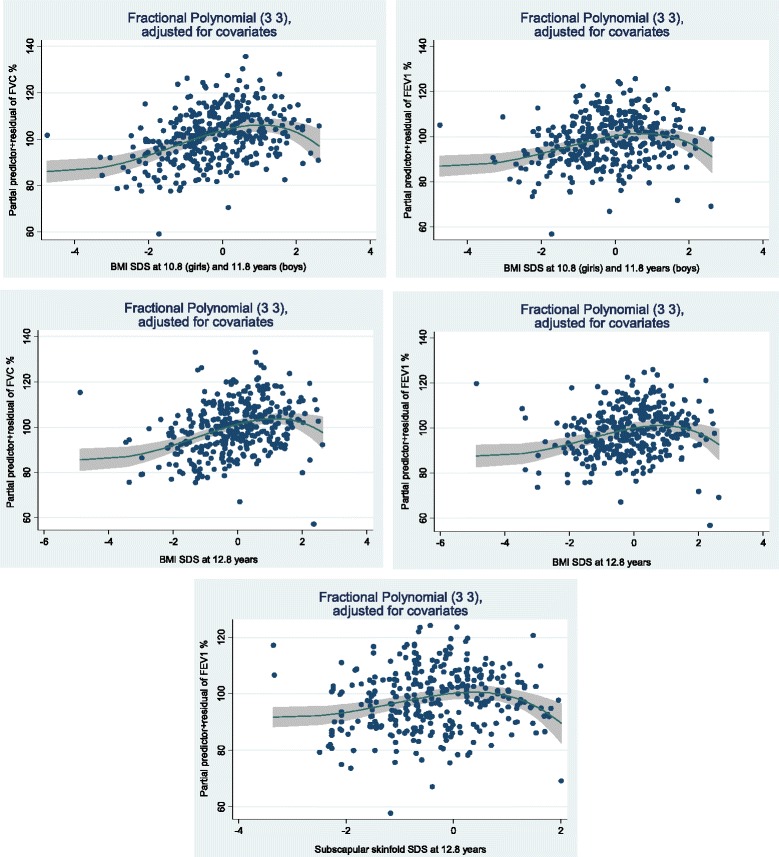
Fractional polynomial residual plots of lung function measures versus BMI SDS and subscapular skinfold SDS. FVC % = forced vital capacity in percent of predicted. FEV_1_% = forced expiratory volume in first second in percent of predicted. BMI = body mass index. SDS = standard deviation score. The numbers in the brackets in each figure are the powers in the fractional polynomial (FP) with the best fit for each measure (X), i.e. FP(3,3) = b_1_X^3^ + b_2_X^3^∙ln(X). The plots show the predicted values of the response variables given the categorical covariates set to their reference value (0) and the other continuous variables to their mean values, i.e. sex = female, gestational age at birth = 39.2 weeks, birth order = not firstborn, breastfeeding = none, mother’s preeclampsia = no preeclampsia, mother’s BMI = 23.6, mother’s smoking = no, mother’s asthma = no, mother’s education = <9 years, and mother’s age at delivery = 28.2

Other anthropometrics were positively associated with FVC % and FEV_1_%, but interaction analyses showed that for most variables this was only found in girls. Several of these variables were negatively associated with FEV_1_/FVC % and FEF_25–75%_/FVC, without differences between genders (Table 4).

Change in BMI SDS from 1 to 4 years was positively associated with FVC % and FEV_1_%. Change in BMI SDS from the age of 4 to 10.8/11.8 years (girls/boys) was positively (significant or near significant) associated with FVC % and FEV_1_% and negatively associated with FEV_1_/FVC % and FEF_25–75%_/FVC (Table 5).

In the MFPR analyses, significant inverse U-shaped associations were found between BMI SDS at the age of 10.8/11.8 (girls/boys) and FVC % and FEV_1_%, but after stratification for gender, these associations were found only for girls. Inverse U-shaped associations were also found between BMI SDS at the age of 12.8 years (both genders) and FVC % and FEV_1_% and between subscapular skinfold at 12.8 years of age and FEV_1_% (Figure 2). The functions showed a linear increase until a deflection point approximately at BMI of 1 SDS. No other non-straight-line associations were found between anthropometric measures and lung function.

## Discussion

In this retrospective cohort study from birth to 12.8 years of age, after adjustment for confounders, weight-related anthropometrics through childhood were positively associated with FVC % and FEV_1_% at 12.8 years of age. Different weight related anthropometrics in late childhood and change in BMI from early to late childhood were negatively associated with FEV_1_/FVC % and FEF_25–75%_/FVC at 12.8 years of age. BMI SDS at the age of 10.8/11.8 years (girls/boys) and 12.8 years (both genders) had an inverse U-shaped association with FVC % and FEV_1_%, potentially indicating that an increase in BMI over a given threshold adversely influences lung function. For the anthropometric variables other than BMI, the positive associations with lung function were generally only found for girls.

The positive associations of anthropometric measures with FVC and FEV_1_ and the negative associations with FEV_1_/FVC and FEF_25–75%_/FVC at 12.8 years of age are consistent with the results from several cross sectional [7–10] and longitudinal studies in children [3, 12, 13, 36]. Similar results were found in a recent meta-analysis of 25,000 children from 24 European birth cohorts [37], but the age of lung function and anthropometric measurements differs between studies [3, 7, 9, 10, 12, 36, 37].

We found positive overall straight-line associations for birthweight and accelerated weight gain at the ages from 1 to 4 years and from 4 to 10.8/11.8 years (girls/boys) with lung function at 12.8 years of age. This is in accordance with another study reporting a positive association both between rapid weight gain from 3 to 7 years of age and FVC and FEV_1_ at 15 years of age [12]. The positive associations between changes in BMI with lung function, both at the ages of 1–4 years and 4 to 10.8/11.8 years (girls/boys), were in contrast to the results from a recent published study of 1740 Australian children followed from birth to 21 years of age [38]. The authors found that catch-up growth during the first 5 years of life had a positive effect on lung function at 21 years of age, whereas an inverse effect on lung function was found in men if the weight gain or obesity started after 5 years of age [13]. Differences in results between studies may be due to different predictors and ages of anthropometric measurements.

The reasons for the associations between anthropometrics and lung function found by us and others are not clear. Within a normal BMI range, it may seem that accelerated weight gain through childhood is associated with both increased lung volumes (FVC) and airway size (FEV_1_), but more so for lung volumes [7, 12, 37]. This disproportionate growth between lung size and airway calibre during childhood has been called ‘dysanapsis’, by many considered a physiological phenomenon and without clinical relevance for airflow limitation in individuals [39, 40]. However, the lower FEV_1_/FVC ratio related to BMI and weight gain in childhood has by some authors been interpreted as a flow limitation [10, 36, 39]. Recently, in a publication including 6 cohorts of children, dysanapsis defined as a normal FVC and FEV_1_ and a low FEV_1_/FVC ratio was associated with disease severity in obese children with asthma [41].

Asthma may be related to lung function, but is a heterogeneous disease and in clinical practice not a diagnosis with highly specific criteria. Furthermore, the direction of possible causality between child’s asthma and anthropometrics and/or lung function may go either way, and asthma was not included as a potential confounder. We found no considerable difference in lung function between those children who did and did not report asthma. In a previously published paper of the same cohort, we found no association between childhood anthropometrics and asthma at 12.8 years of age [27], consistent with an Italian cross-sectional study of 2393 children aged 10–17 years, the negative association between weight and FEV_1_/FVC was independent of wheeze or other respiratory symptoms [7]. In contrast, two large scale studies have shown positive associations between rapid weight gain during early childhood and asthma and wheeze in later childhood [12, 42]. Consistently, in a meta-analysis including 1.4 million participants aged 0–19 years, overweight and obesity were positively associated with childhood asthma [16].

The pattern of associations between early-life anthropometrics and lung function discussed above is in contrast to equivalent studies in adults, showing negative associations between different anthropometrics and both FVC and FEV_1_ [5, 6]. A study including longitudinal data concluded that an initial positive association between BMI in childhood and adult FVC and FEV_1_ was likely to be attributable to greater childhood lean body mass and not greater fat mass alone [5]. BMI in normal-weight children is more a surrogate marker of lean body mass than fat mass, suggesting that muscle mass increases in parallel with BMI; this could explain the different results in children and adults [18]. Skinfolds and waist circumference may be better markers of adiposity than BMI in children [19–21]. However, we found equivalent associations of BMI, waist circumference and skinfolds with lung function variables, which may suggest that BMI also reflects body fat in these children, and it may contradict the interpretation that the association between childhood BMI and lung volumes is only a result of lean body mass.

The positive associations between anthropometric variables other than BMI and FVC % and FEV_1_% were mainly found in girls, which is consistent with other studies. In a study of 1583 12-year old Canadian children, Khan et al. found that in boys, adiposity was negatively associated with lung function, but not in girls [8]. In a follow up of 11-year old children, Wang et al. found that BMI was positively associated with lung function only in girls, whereas body fat mass was negatively associated with lung function only in boys [43]. These gender specific differences could be due to differences in fat accumulation during and after puberty, with a peripheral fat deposition in girls and an abdominal fat deposition in boys, leading to reduction in lung function in boys [8].

The gender differences in the association between other anthropometric measurements than BMI and FVC % and FEV_1_% were found only at the second follow-up at 12.8 years of age, except for waist circumference, where the same pattern appeared at the first follow-up at 10.8/11.8 years. This result may support that sex hormones are important determinants not only for anthropometry, but also for lung development [39].

The MFPR analyses in the present study showed inverse U-shaped associations of BMI SDS at the ages of 10.8/11.8 years and 12.8 years with FVC % and FEV_1_% at 12.8 years, but no such associations were found for BMI at younger ages. Figure 2 shows a possible inverse relationship in children with a BMI SDS from slightly above 1 corresponding to IOTF class overweight, in accordance with other studies suggesting that BMI beyond a threshold is associated with reduced lung function [10, 13, 44]. When stratifying by gender, the inverse U-shaped associations persisted only for girls at 10.8 years of age, possibly owing to loss of power. In a Mexican study, an inverse U-shaped association between BMI and FEV_1_ was found only in children older than 12 years of age [44]. In the PIAMA study with measurements of lung function at 8 and 12 years of age, an inverse U-shaped association of BMI and waist circumference with FVC and FEV1 was found, however the effect size was small and only found in boys [10]. The authors suggested that the transition from the childhood association (high BMI associated with larger lung volume) to the adult association (high BMI associated with smaller lung volumes) takes place later than 12 years [10]. Our results indicate that a transition from a positive to a negative association between BMI and lung function may appear earlier than 12 years for children with the highest BMI. This may be the earliest age indicating the negative association between BMI and lung function seen over a broader range of BMI observed in adults [5, 6].

One potential reason for a negative association between obesity and lung volume is a mechanical effect of internal fat deposition, reducing chest wall compliance and impeding diaphragmatic descent [45]. The inverse U-shaped association between subscapular skinfold and FEV_1_% in our study could support this.

A negative association between higher levels of leptin or leptin/adiponectin ratio with lung function could also be mediated by increased systemic inflammation [15], supported by studies showing an association between adiposity and clinical asthma in childhood and adulthood [15, 46].

### Clinical relevance

The positive and negative regression coefficients between the anthropometric measures and lung function in the present study are small, i.e. in the range 1–3. Thus, if the predictors increased by one unit (SDS, which is a substantial increase), the predicted lung function values increased or decreased by 1% to 3%, making the clinical relevance of the results less substantial. However, the inverse U-shaped associations between weight-related anthropometrics and lung function at 10.8–12.8 years of age could suggest that the greater impact of airway obstruction occurs in children with the highest body fat mass.

### Strengths and limitations

The study population was stable and homogeneous regarding socio-economic status and ethnicity. The low participation rate at both follow-ups reduces power, increases the risk of false negative results and may be a source of bias, whereas the loss to follow-up from the first to the second may be a source of selection bias. However, we found no perinatal differences between those who assented to the first follow-up and those who did not assent, nor did baseline characteristics differ between those who participated in the first and second follow-ups.

The study design was longitudinal, but the analyses of the associations between anthropometry and lung function at the second follow-up were cross-sectional. Therefore, reverse causation cannot be excluded. However, as the results were consistent across childhood, reverse causation may be less likely.

This study was originally a follow-up of a nested case-control study of preeclampsia, with one-third of the participants exposed to preeclampsia in utero; thus, the cohort is not representative of a general population. However, preeclampsia was adjusted for in the analyses and did not confound the results. Missing responses from the ISAAC questionnaire were interpreted as negative, and we cannot be sure that a missing response was a true negative response for all children. Furthermore, we adjusted the analyses for physical activity, although the questions about physical activity were only validated for adults.

## Conclusions

In this cohort followed from birth, weight-related anthropometric measures during childhood were positively associated with FVC % and FEV_1_% and negatively associated with FEV_1_/FVC % and FEF_25–75%_/FVC in late childhood. Whereas these findings could result from physiological phenomena, increasing weight might also influence the diameter of the airways contributing to airflow limitation. The inverse U-shaped associations of BMI in late childhood with lung volumes and airways size suggest that the highest impact of BMI on lung function occurs in children with the highest BMI.

## Additional files

Additional file 1: Figure S1. Directed Acyclic Graph. Colours of rings: Green = predictor; blue with black dot = outcome; blue = ancestor of outcome; red = potential confounder; black = adjustment set; grey = unavailable/unknown confounders. Red line = biasing path; green line = causal path; black line = closed path. The figure was made by using DAGitty software. (PDF 41 kb)

## Abbreviations

b: Regression coefficient
BMI: Body mass index (kg/m^2^)
CI: Confidence interval
FEF_25–75%_: Forced expiratory flow at 25–75% of forced vital capacity
FEV_1_: Forced expiratory volume in the first second
FVC: Forced vital capacity
IBM SPSS: International business machines corporation statistical package for the social sciences
ISAAC: International study of Asthma and Allergy in Childhood
MFPR: Multiple fractional polynomial regression
SDS: Standard deviation score
