# Supplementary figures and images for: The associations between weight-related anthropometrics during childhood and lung function in late childhood: a retrospective cohort study
Source: BMC Pulm Med. 2018 Jan 19;18:10. doi: 10.1186/s12890-017-0567-3 (PMC5775530; doi:10.1186/s12890-017-0567-3)

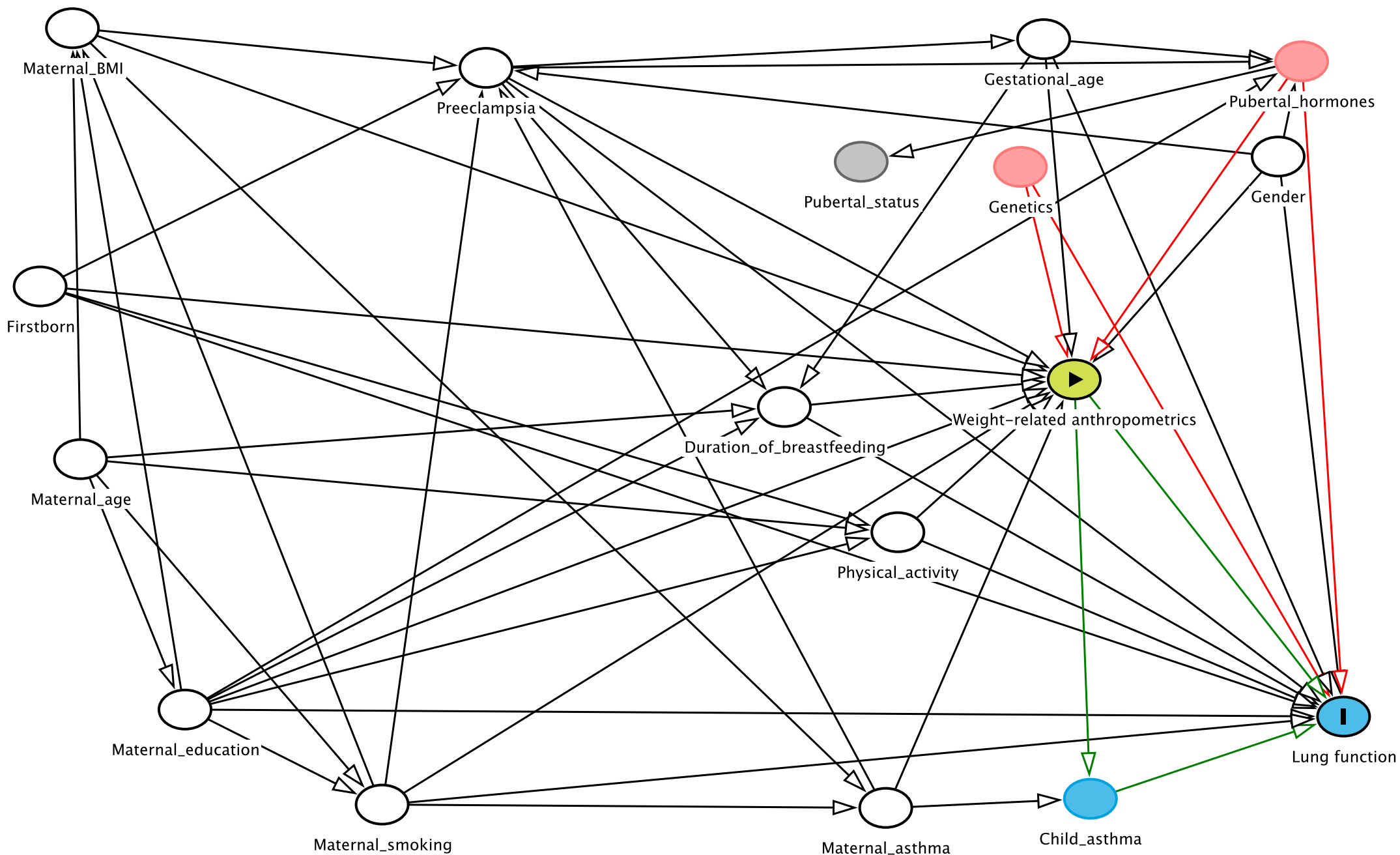

Supplement: Supplementary file 1 — Directed Acyclic Graph. Colours of rings: Green = predictor; blue with black dot = outcome; blue = ancestor of outcome; red = potential confounder; black = adjustment set; grey = unavailable/unknown confounders. Red line = biasing path; green line = causal path; black line = closed path. The figure was made by using DAGitty software. (PDF 41 kb) [file 12890_2017_567_MOESM1_ESM.pdf]
